# Cost–effectiveness analysis of quadrivalent influenza vaccine in Spain

**DOI:** 10.1080/21645515.2016.1182275

**Published:** 2016-05-16

**Authors:** Amos García, Raúl Ortiz de Lejarazu, Jordi Reina, Daniel Callejo, Jesús Cuervo, Raúl Morano Larragueta

**Affiliations:** a Servicio Epidemiología y Prevención, DG Salud Pública Servicio Canario de Salud, Las Palmas, Spain; b National Influenza Center, Servicio Microbiología Hospital Clínico Valladolid, Valladolid, Spain; c Centro de Referencia de la Gripe, Servicio Microbiología Hospital Universitario Son Espases, Palma de Mallorca, Spain; d BAP Health Outcomes Research, Oviedo, Spain; e Dpto. Evaluación de Medicamentos, GSK, Madrid, Spain

**Keywords:** costs and cost analysis, cost-effectiveness analysis, human, healthcare costs, influenza, influenza B virus, influenza vaccines, QIV, vaccines

## Abstract

Influenza has a major impact on healthcare systems and society, but can be prevented using vaccination. The World Health Organization (WHO) currently recommends that influenza vaccines should include at least two virus A and one virus B lineage (trivalent vaccine; TIV). A new quadrivalent vaccine (QIV), which includes an additional B virus strain, received regulatory approval and is now recommended by several countries. The present study estimates the cost-effectiveness of replacing TIVs with QIV for risk groups and elderly population in Spain. A static, lifetime, multi-cohort Markov model with a one-year cycle time was adapted to assess the costs and health outcomes associated with a switch from TIV to QIV. The model followed a cohort vaccinated each year according to health authority recommendations, for the duration of their lives. National epidemiological data allowed the determination of whether the B strain included in TIVs matched the circulating one. Societal perspective was considered, costs and outcomes were discounted at 3% and one-way and probabilistic sensitivity analyses were performed. Compared to TIVs, QIV reduced more influenza cases and influenza-related complications and deaths during periods of B-mismatch strains in the TIV. The incremental cost-effectiveness ratio (ICER) was 8,748€/quality-adjusted life year (QALY). One-way sensitivity analysis showed mismatch with the B lineage included in the TIV was the main driver for ICER. Probabilistic sensitivity analysis shows ICER below 30,000€/QALY in 96% of simulations. Replacing TIVs with QIV in Spain could improve influenza prevention by avoiding B virus mismatch and provide a cost-effective healthcare intervention.

## Introduction

Seasonal influenza is an acute viral infection that circulates worldwide and spreads easily from person to person. It can affect any age group, cause annual epidemics and represents a serious public health problem, due to the severity of the illness and the number of deaths in high risk populations. Furthermore, influenza can have a huge economic impact through reduced workforce productivity and overwhelmed health services during peaks in infection.^1^

There are three types of seasonal influenza viruses: A, B and C. Type C influenza cases occur much less frequently than types A and B^1^ but type A influenza viruses cause most influenza infections. Nevertheless, type B infection is also frequent in children and young adults and is the predominant virus to cause epidemics every 2–4 y.^2^ Type A and B infections produce similar clinical symptoms, hospitalization rates and rates of admission to Intensive Care Units.^3,4^

Influenza vaccination is the most effective way to prevent infection and thereby disease development and potential severe outcomes. A number of safe and effective vaccines are available and have been used for more than 60 y.^1^ Current available trivalent vaccines (TIVs) protect against two A subtypes (H1, H3) and one B lineage. However, as global co-circulation of two B lineages has occurred in the past, there remains a gap to be filled.^2,5^ The proportion of influenza infections that are not covered with TIVs varies from year to year due to B type mismatch between vaccine and circulating B lineages. In Spain, in seven out of eight seasons since 2005/2006 (excluding the pandemic influenza season in 2009/2010), two distinct B lineages have co-circulated. As a result of this mismatch, the TIVs have not been fully adequate during the last eight seasons. A quadrivalent inactivated influenza vaccine (QIV) has therefore recently been developed to address the unmet need of adequate protection in case of mismatch between circulating B viruses.

The National Immunization Schedule in Spain is the calendar that defines the antigens and schedules (including recommended number of doses and ages) for the systematic vaccination of the entire population. There is a framework for the systematic assessment of any changes to the schedule (e.g. the inclusion of new antigens or modifications to current regimens). This considers five assessment criteria: burden of disease, efficacy and safety, impact of change in the vaccination schedule (i.e. co-administration issues), ethical considerations and economic evaluation.^6^

The economic evaluation of health technologies is defined as the comparative analysis of alternative courses of action in terms of both their costs and their consequences.^7^ Together with other relevant criteria, such evaluations provide useful knowledge to facilitate informed healthcare resource allocation decisions.

The aim of the present study was to compare the cost-effectiveness of vaccination programs in Spain with either TIV or QIV in preventing seasonal influenza in elderly (≥ 65 y old) and at risk individuals (≥ 3 y old).

## Results

### Base case analysis (lifetime horizon)

Over a lifetime horizon (100 y) evaluation of influenza vaccinated age-cohorts, using QIV would result in 40,000 additional quality-adjusted life years (QALYs) gained compared to the use of TIV, with an increased cost of 350 million €. The incremental cost-effectiveness ratio (ICER) of QIV over TIV was 8,748€/QALY gained (Table 1). From the National Health System (NHS) perspective, costs related to the use of QIV are higher mainly because of the difference in vaccine price versus TIV. That difference is however offset when considering societal costs, due to the lower QIV indirect costs (less productivity loss and absenteeism).

**Table 1.** Cost-effectiveness of quadrivalent influenza vaccination (QIV) compared to trivalent vaccine (TIV): base case scenario with lifetime horizon.

	TIV	QIV	Difference
Spanish Healthcare System costs	11,901,394,637 €	12,348,949,428 €	447,554,791 €
Societal costs	34,462,064,137 €	34,364,483,970 €	− 97,580,167 €
Total costs	46,363,458,774 €	46,713,433,398 €	349,974,624 €
Life years	1,143,182,206	1,143,233,538	51,332
QALYs	1,038,585,055	1,038,625,059	40,005
ICER (€/QALY) NHS perspective	11,188 €		
ICER (€/QALY) Societal perspective	8,748 €		

TIV: Trivalent influenza vaccine; QIV: Quadrivalent influenza vaccine; QALY: Quality-adjusted life year; ICER: Incremental cost-effectiveness ratio; NHS: National Health System.

### One-year time horizon results

Although the lifetime horizon allows a holistic evaluation of the value of QIV in the long term, a short term analysis would be better for estimating the potential health impact of QIV.

Using a one-year scenario (year after administration was initiated), and average seasonal matching data, the preventive strategy of influenza vaccination with QIV would deliver significant reductions in both disease-related morbidity and mortality: 18,565 influenza cases; 2,577 influenza-related complications; 407 influenza-related hospitalizations and 181 deaths compared to TIV during the first year; with an incremental cost of 11,203,359€ due to the incremental vaccine cost (17.7 million €) partially offset by cost savings in absenteeism (−3.7 million €); influenza complications (−2.2 million €) and uncomplicated influenza (−0.4 million €).

**Figure 1.**
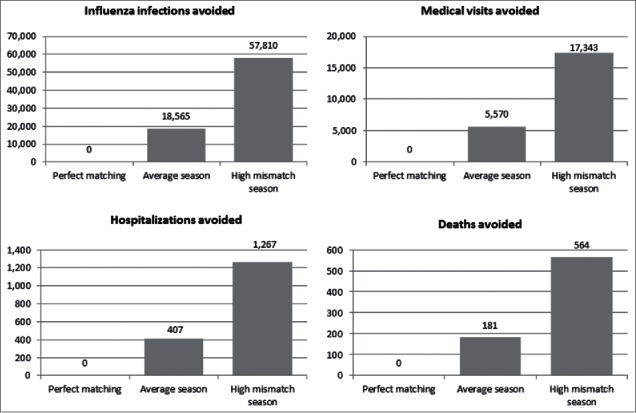
Additional outcomes of quadrivalent influenza vaccine (QIV) versus trivalent influenza vaccine (TIV) at one-year time horizon *Legend* Perfect matching: 100% matching between B strain circulating and B strain included in TIV; Average season: mean B strain matching over the last 8/10 seasons; High mismatch season: data from 2007–2008 season, with high mismatch between B strain included in trivalent influenza vaccine and B strain circulating.

If we consider a mismatched season, the outcomes were more greatly improved. Figure 1 shows the annual outcomes of QIV depending on the match achieved by the vaccine.

### Sensitivity analysis

The one-way sensitivity analysis found that the parameters most affecting the cost-effectiveness of QIV were: the circulation of type A influenza (higher circulation of A virus resulting in a lower differential benefit for QIV) and the potential mismatch between the circulating type B lineage and the one included in TIV (higher mismatch leading to better cost-effectiveness for QIV). Obviously, if there was almost insignificant type B circulating influenza or if lineage B matching was perfect, QIV would not offer any additional benefit over TIV. None of the other tested parameters produced meaningful changes on the results.

**Figure 2.**
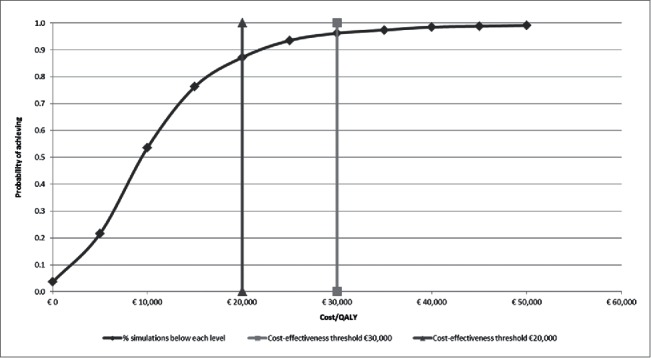
Probabilistic sensitivity analysis, cost-effectiveness acceptability curve *Legend* QALY: Quality-adjusted life year.

The probabilistic sensitivity analysis, including a total of 1,000 simulations, showed that in 3.6% of simulations, QIV achieved better health outcomes than TIV with lower total costs (dominant). At a willingness to pay threshold of 20,000€/QALY, QIV had an 87% probability to be a cost-effective alternative for influenza prevention as compared to TIV; this rose to 96% at a willingness to pay threshold of 30,000€/QALY (Fig. 2).

### Discussion

Cost-effectiveness analysis should be an additional tool that, together with epidemiological and clinical evidence, contributes for discussing about inclusion of new antigens or expand vaccination programs.

This analysis used a lifetime, multi-cohort Markov model to compare the potential effects of QIV and TIV on the disease burden of influenza in Spain from the societal perspective. Our base case results indicated that the QIV would deliver substantial health benefits from the NHS perspective by further reducing the number of symptomatic influenza cases, of medical visits, of hospitalizations for complications, and of deaths as well as of work absenteeism, compared to TIV. The estimated reduction in influenza cases with QIV would also reduce the costs of treating influenza and indirect costs resulting from time lost from work due to influenza, partially offsetting the increased costs of QIV compared to TIV. Overall, QIV was estimated to be a cost-effective intervention compared to TIV, with an ICER estimated at 8,748€/QALY.

The results of the study are consistent with findings from studies in the USA and the UK, which indicated that QIV would be expected to reduce influenza cases, hospitalizations and deaths, more than TIV.^8,9^ It is also aligned with other vaccines cost-effectiveness analyses targeting adult and elderly, like pneumococcal and zoster vaccination, with the difference that influenza needs to be addressed every year while the others require one-time vaccination and no recurrent changes in vaccine composition.

A lifetime model, such as the one reported in this article, can follow a cohort of individuals over a lifetime of influenza seasons and repeated vaccination and/or other interventions. A lifetime cohort model is a better option than a one-year model to answer research questions about the cost-effectiveness of a particular vaccination policy when applied to today's eligible population cohorts, who will then age over time. Due to differences in modeling approaches, the results of this model are not directly comparable with previously published results from one-year models.^10,11^

A multi-cohort model could reflect population heterogeneity. Different age groups may vary in their probability of infection, baseline utility, mortality risk and other factors. However, this capacity for heterogeneity was not completely taken advantage of, because detailed age-specific data were not available. However, some inputs, such as length of stay for the complications that required hospitalization, exploited this granularity. This model structure allows granular information to be included as soon as it becomes available. However we recommend that a model selection should be based on the research question to answer rather than being determined by data availability.^12^

A number of limitations arise from this study. Firstly, as the model adopted the Ministry of Health recommendations for influenza vaccination,^13^ the population targets for vaccination were the elderly over 65 y and the at-risk groups below 65 y. At a regional level, there could be different target populations for influenza vaccination and the model could not therefore represent an exact picture across Spain. Nevertheless, the Spanish Health Authorities are now trying to introduce a unique vaccination calendar for the whole country, unifying recommendations, and therefore the information used in the model would be more representative.

Secondly, herd effect is not included in this analysis. Several studies have shown how indirect herd effect from vaccination of children offers potential for improving the effectiveness of influenza prevention in the remaining unvaccinated population. For example, a study in Canada found that vaccination against influenza of children and adolescents up to the age of 15 y achieved a protection of 61% against influenza infection in unvaccinated individuals.^14^ However, that effect cannot be captured by static models like the present Markov model. Further dynamic modeling approaches are needed to explore herd effect impact of influenza vaccination. Not including herd effect in the present study was nevertheless a conservative assumption against QIV, which would be expected to have a higher herd effect due to its additional B virus strain. Including a herd effect, as estimated from influenza studies,^15^ would result in a more favorable ICER for QIV.

Obviously it is not possible to predict influenza virus circulation over the next 100 years, it is already difficult enough to predict the circulation for the next season. We used an average from the latest seasons of type B influenza circulation and matching B lineage to model future circulation over a 100 y time horizon. Seasonal influenza variations and the unpredictability of the influenza virus in the future may prove that estimations from historical data could be incorrect. However, at the time of the analysis, they were considered the best available estimates.

Finally, there were limitations in the data available to populate the model. Often there was a lack of data regarding the differences between healthy and at risk groups. Consequently, some data included in the model were taken from studies conducted outside Spain. Spanish data were preferred over studies conducted abroad, but if no Spanish data were available or they were considered to be unrepresentative due to small sample sizes, other European sources were chosen.

In conclusion, this economic evaluation of QIV compared to TIV in elderly and clinical risk groups in Spain, using a multi-cohort Markov model, estimated that QIV will further reduce influenza cases, complications, hospitalizations, and deaths compared to TIV. With a cost of 8,748€/QALY gained, it would provide a cost-effective intervention at the 30,000€/QALY threshold in Spain.^16,17^ Including QIV within national immunization programs could therefore contribute to reduce the burden of disease and alleviate the huge healthcare demand that occurs every year during the influenza season.

## Methods

A static, lifetime, multi-cohort state transition model with a one-year cycle time, which had been previously used to assess QIV in the UK,^8^ was adapted to the Spanish setting. Nine age groups were considered: 0–4, 5–17, 18–49, 50–64, 65–69, 70–74, 75–79, 80–84 and ≥ 85 years, which were split in 2 groups according to risk: the first group included the population who was at risk of serious complication from influenza due to other conditions and chronic diseases (“*at-risk group*”). The second group included a healthy population for whom the influenza vaccine is also recommended, for example individuals aged over 65 y (“*healthy group”*). The at-risk group was defined according to the Spanish Ministry of Health recommendations for influenza vaccination,^13^ and included patients with chronic cardiovascular or lung disease, metabolic disease, morbid obesity, chronic renal disease, hemoglobin disorders and anaemia, asplenia, chronic liver disease, severe neuromuscular diseases, immunosuppressed, cochlear implanted, cognitive dysfunction, people living in closed institutions, pregnant women and children from 6 months to 18 y receiving long-term treatment with acetylsalicylic acid. For the age group 0–4 y, the proportion of vaccinated subjects was adjusted according to QIV approved indication (children >3 y old only are vaccinated).^18^

The vaccine coverage rate was adjusted for each age and risk group to calculate the number of vaccinated individuals. A lifetime horizon (100 y) was considered to allow a comprehensive analysis of the clinical benefits in terms of accrued life years (LYs) and QALYs. Hence, the model allowed cases in the youngest age group to be followed up through all age groups. Once an age cohort reached the starting age of the next age cohort, all probabilities of the corresponding age group were applied.

Influenza infection was split according to virus type A (H1N1 and H3N2 together) and type B (Victoria and Yamagata separately) to ensure that additional QIV protection benefit could be captured.

As influenza causes annual epidemics and yearly vaccination is recommended, a one-year cycle was chosen. A number of events could happen during each cycle, with any subject having a differential probability of the following events: vaccination; suffer influenza infection; seek medical advice for influenza (Primary Care or Emergency Room); suffer influenza-related complications; need hospitalization for complication; death. All survivors from each cycle would begin a new annual cycle.

Two prevention strategies were analyzed: influenza vaccination with either QIV or TIV.

The economic evaluation of QIV was conducted from a societal perspective, which allowed the costs associated with sick leave to be included, and also from the NHS perspective.

The model was developed in *Microsoft Excel 2010*. A discount rate of 3% was used for costs and health outcomes as recommended.^19^ All costs were in 2014 euros.

### Inputs of the Economic Model

#### Demographic data

Details on the Spanish population (46,727,891 individuals), and their distribution by age, risk groups and all-causes mortality rates were obtained from the National Statistics Institute and National Health Survey 2011–2012.^20-23^ Several assumptions were required due to a lack of information. Based on UK data,^24^ the model assumed that all-cause mortality in the at-risk group was 10-fold the all-cause mortality in the healthy group. The probability of moving to the at-risk group was independent of influenza exposure or vaccination status, and was calculated from all-cause mortality data and the age distribution of the at-risk group. Once individuals moved to the at-risk group, they remained in this group for the remainder of their life.

#### Vaccine efficacy

Vaccine efficacy against influenza type A was assumed to be the same for QIV and TIV, and was estimated differentially for age groups based on three Cochrane systematic reviews in healthy children,^25^ healthy adults,^26^ and the elderly.^27^ Efficacy in the at-risk groups was assumed to be identical to that of healthy groups.

The efficacy of TIV against influenza type B was estimated from a meta-analysis in adults which considered the case of perfect matching and total mismatching.^28^ A reduction of efficacy in children and the elderly was applied.^25,28,29^ Notably, TIV does have some cross-protection against type B influenza in the case of mismatching.^28^ Efficacy against type B influenza is the main difference between TIV and QIV, due to the inclusion of the second type B lineage in QIV, which increases the probability of matching with the circulating type B influenza. It is assumed that TIV efficacy against influenza B is proportional to the percentage of matching with circulating type B influenza. Hence, QIV efficacy would be equal to the efficacy of TIV in the event of optimal matching.

The proportion of circulating type B influenza within all influenza cases (type A + type B), which varies by year, also has an effect on the incremental health benefit of QIV over TIV. The model estimates type A and type B circulation based on the average of the last ten influenza seasons in Spain, excluding the season of pandemic influenza 2009–2010, provided by Spanish National Epidemiology Center.^30-39^ This source was also used to determine the average matching between circulating type B lineage and the one included in TIV, over the last eight seasons, excluding the pandemic season. Table 2 summarizes influenza circulation and matching data in Spain.

**Table 2.** Influenza circulation, lineage and matching in Spain.^30-39^

	B lineage circulation				Proportion of predominant virus	
Season	Victoria	Yamagata	B lineage in trivalent vaccine	Mismatching B	Type A	Type B
2003–2004					99.75%	0.25%
2004–2005					83.78%	16.22%
2005–2006	86.4%	13.6%	Yamagata	86.4%	59.61%	40.39%
2006–2007	11.1%	88.9%	Victoria	88.9%	90.92%	9.08%
2007–2008	3.0%	97.0%	Victoria	97.0%	47.00%	53.00%
2008–2009	100.0%	0.0%	Yamagata	100.0%	73.00%	27.00%
2010–2011	95.6%	4.4%	Victoria	4.4%	72.12%	27.88%
2011–2012	15.4%	84.6%	Victoria	84.6%	92.38%	7.62%
2012–2013	17.2%	82.8%	Yamagata	17.2%	25.23%	74.77%
2013–2014	35.3%	64.7%	Yamagata	35.3%	99.10%	0.90%
Average	45.5%	54.5%		64.2%	74.29%	25.71%

#### Probabilities

Vaccination coverage was estimated from the 2011–2012 National Health Survey for adults over 18 y,^23^ and from a regional population study for children.^40^ It was assumed that vaccination followed Ministry of Health recommendations. Other assumptions were: equal vaccination coverage would be reached with TIV and QIV, and QIV was used according to the label age indications (≥ 3 y).^18^

The annual probability of developing symptomatic influenza without prophylaxis is reportedly higher in children (19.21%) than in adults (6.55%) or the elderly (6.17%).^5^ The probability of seeking medical advice (Primary Care or Emergency Room) for influenza symptoms was estimated at 30% in Spain, based on a volunteer internet-registry to monitor the activity of influenza-like illness.^41^ The proportion of population seeking advice at the Primary Care (81.67%) or Emergency Room level was established according to clinical expert opinion.

Neuraminidase inhibitors are not reimbursed in outpatient settings in Spain, and they are not usually prescribed for post-exposure prophylaxis or influenza treatment. This Spanish economic evaluation does not therefore include the use of neuraminidase inhibitors.

The probabilities of developing influenza complications, type of complication, hospitalization, and death related to influenza, were taken from a large UK study.^42,43^ This source was used because the information available in Spain came from small studies not representatives of the whole population.^44,45^

Table 3 shows a summary of demographic, efficacy and probability inputs.

**Table 3.** Demographic, efficacy and probability inputs used to populate the model.

Age group (years)	0–4	5–17	18–49	50–64	65–69	70–74	75–79	80–84	85+
Population distribution	5.18%	12.73%	46.04%	18.37%	4.86%	3.67%	3.69%	2.93%	2.53%
Proportion healthy in each age group	81.18%	78.63%	68.63%	36.18%	21.21%	12.72%	11.60%	10.13%	9.13%
Population at-risk in each age group	18.82%	21.37%	31.37%	63.82%	78.79%	87.28%	88.40%	89.87%	90.87%
Vaccine efficacy against influenza A, trivalent and quadrivalent	59.00%	59.00%	60.00%	60.00%	58.00%	58.00%	58.00%	58.00%	58.00%
Trivalent vaccine efficacy against influenza B, match	66.00%	77.00%	77.00%	73.00%	69.00%	69.00%	66.00%	66.00%	66.00%
Trivalent vaccine efficacy against influenza B, mismatch	44.00%	52.00%	52.00%	49.00%	47.00%	47.00%	44.00%	44.00%	44.00%
Trivalent vaccine efficacy against influenza, base case	51.87%	60.95%	60.95%	57.59%	54.87%	54.87%	51.87%	51.87%	51.87%
Quadrivalent vaccine efficacy against influenza B	66.00%	77.00%	77.00%	73.00%	69.00%	69.00%	66.00%	66.00%	66.00%
Influenza vaccine coverage, healthy	0.00%	0.00%	0.00%	0.00%	28.38%	49.55%	48.18%	64.56%	57.63%
Influenza vaccine coverage, at-risk	24.16%	24.24%	9.26%	24.54%	47.00%	54.40%	63.85%	72.47%	67.59%
Influenza-related complication, healthy	14.05%	14.05%	7.61%	7.95%	10.34%	10.34%	10.34%	10.34%	10.34%
Influenza-related complication, at risk	18.29%	18.29%	12.32%	12.59%	13.76%	13.76%	13.76%	13.76%	13.76%
Hospitalization due to complication, healthy	10.87%	10.87%	10.87%	10.87%	15.79%	15.79%	15.79%	15.79%	15.79%
Hospitalization due to complication, at risk	15.79%	15.79%	15.79%	15.79%	15.79%	15.79%	15.79%	15.79%	15.79%
Death after influenza complication, healthy	0.00%	0.00%	0.405%	0.96%	11.21%	11.21%	11.21%	11.21%	11.21%
Death after influenza complication, at risk	0.15%	0.15%	0.34%	1.64%	12.18%	12.18%	12.18%	12.18%	12.18%

#### Costs

Unitary costs applied to health resource consumption were retrieved from Spanish cost databases and official tariffs published by health authorities^46-52^ and from previously published studies of indirect costs associated with influenza.^53^ When the same costs were found from different sources, an average cost was considered. The wholesale price of the vaccines was considered and the administration costs were taken from regional tariffs.^46-52^ The remaining unitary costs were equal for both alternatives.

The analysis was conducted from a societal perspective and therefore included both direct medical costs and costs incurred by the patient or society. Absenteeism costs and productivity loss caused by influenza in the working-age population (18–65 y) was estimated based on National Statistics Institute data. A complete list of all unitary costs included in the model is available in Table 4.

**Table 4.** Unitary costs.^46-53^

Age group (years)	0–4	5–17	18–49	50–64	65–69	70–74	75–79	80–84	85+
**National Health System Costs**									
Quadrivalent vaccine	9.50 €	9.50 €	9.50 €	9.50 €	9.50 €	9.50 €	9.50 €	9.50 €	9.50 €
Trivalent vaccine	7.00 €	7.00 €	7.00 €	7.00 €	7.00 €	7.00 €	7.00 €	7.00 €	7.00 €
Vaccine administration	11.00 €	11.00 €	11.00 €	11.00 €	11.00 €	11.00 €	11.00 €	11.00 €	11.00 €
Primary Care visit	52.73 €	52.73 €	52.73 €	52.73 €	52.73 €	52.73 €	52.73 €	52.73 €	52.73 €
Emergency Room	127.35 €	127.35 €	146.10 €	146.10 €	146.10 €	146.10 €	146.10 €	146.10 €	146.10 €
Outpatient complication	35.18 €	35.18 €	35.74 €	35.74 €	35.74 €	35.74 €	35.74 €	35.74 €	35.74 €
Bronchitis hospitalization	3,261.84 €	3,274.08 €	2,933.41 €	2,877.61 €	2,786.57 €	2,724.34 €	2,662.10 €	2,680.25 €	2,690.46 €
Pneumonia hospitalization	3,373.31 €	4,082.09 €	5,730.34 €	9,377.09 €	9,241.09 €	9,120.06 €	9,489.30 €	4,723.53 €	4,018.12 €
URTI hospitalization	3,373.31 €	4,082.09 €	5,730.34 €	9,377.09 €	9,241.09 €	9,120.06 €	9,489.30 €	4,723.53 €	4,018.12 €
Hospitalization for cardiac complication	4,283.24 €	3,530.25 €	6,660.38 €	5,106.92 €	4,886.06 €	4,758.99 €	4,501.25 €	4,078.34 €	3,994.09 €
Hospitalization for renal complication	5,344.98 €	4,893.87 €	4,471.18 €	4,150.43 €	3,720.80 €	4,044.61 €	5,219.13 €	3,985.55 €	4,074.83 €
CNS hospitalization	2,602.31 €	2,746.39 €	3,344.55 €	3,131.91 €	4,111.21 €	3,211.37 €	3,808.75 €	3,284.81 €	3,492.01 €
OM hospitalization	2,511.82 €	2,728.14 €	2,648.37 €	2,755.52 €	2,762.39 €	2,879.33 €	2,483.54 €	2,318.71 €	2,196.13 €
**Society cots**									
Productivity loss, influenza	–	–	623.96 €	623.96 €	–	–	–	–	–
Productivity loss, hospitalization	–	–	1,482.70 €	1,482.70 €	–	–	–	–	–
Productivity loss, outpatient complication	–	–	623.96 €	623.96 €	–	–	–	–	–

URTI: upper respiratory tract infection; CNS: central nervous system; OM: otitis media.

#### Health outcomes / utilities

The utilities and disutilities are used for estimating quality of life of subjects suffering from influenza and are needed to calculate QALYs gained through vaccination and thus establish the corresponding cost-effectiveness. Utilities data used in the model were taken from 2011–2012 National Health Survey and were estimated from the EQ-5D^54^ according to age and risk group.^23^ The EQ-5D is a standardized generic instrument that comprises five dimensions: mobility, self-care, usual activities, pain/discomfort and anxiety/depression. Each dimension has one specific question and three levels of response: 1 “no problems,” 2 “some problems” and 3 “severe problems.” The instrument therefore defines distinct health states from all the possible combinations of dimensions and levels of severity. Considering the responses to the descriptive system, each health state is converted into a utility/disutility index by applying the general population preference values. The EQ-5D utility index ranges from 1 (best health status) to negative values (health states valued as worse than death), where 0 is equal to death. This utility index can then be used to calculate QALYs. In the study, disutility caused by influenza infection was retrieved from a large Spanish observational longitudinal study.^55^

Table 5 summarizes the utilities included in the model and the duration of influenza episodes.

**Table 5.** Utility and disutility scores used in the model.^54-55^

Age group (years)	0–4	5–17	18–49	50–64	65–69	70–74	75–79	80–84	85+
**Baseline**									
Healthy	0.99	0.99	0.97	0.96	0.97	0.94	0.93	0.85	0.73
At risk	0.96	0.96	0.94	0.87	0.85	0.82	0.78	0.69	0.54
**Influenza**									
Disutility	− 0.41	− 0.41	− 0.465	− 0.36	− 0.32	− 0.32	− 0.32	− 0.32	− 0.32
Length (days)	7.5	7.5	7.5	7.5	7.5	7.5	7.5	7.5	7.5
**Outpatient complications**									
Disutility	− 0.41	− 0.41	− 0.465	− 0.36	− 0.32	− 0.32	− 0.32	− 0.32	− 0.32
Length (days)	5.4	5.4	5.4	5.4	5.4	5.4	5.4	5.4	5.4
**Inpatient complications**									
Disutility	− 0.54	− 0.54	− 0.60	− 0.58	− 0.56	− 0.56	− 0.56	− 0.56	− 0.56
Bronchitis length	3.49	3.81	5.25	5.70	6.21	6.56	6.49	6.61	6.59
Pneumonia length	4.75	5.94	8.75	10.44	13.55	12.65	14.13	9.33	9.33
URTI length	3.93	3.94	4.50	7.89	5.89	7.85	5.29	6.90	6.62
Cardiac length	10.64	4.75	9.07	9.14	9.07	9.07	8.86	8.06	7.28
Renal length	4.50	4.38	5.03	3.96	5.04	6.91	8.00	7.27	9.81
CNS length	3.46	3.23	4.89	5.68	5.68	6.20	7.85	6.49	6.32
OM length	3.80	2.32	1.93	2.23	2.46	4.04	4.52	5.33	7.40

URTI: upper respiratory tract infection; CNS: central nervous system; OM: otitis media.

#### Analysis

The base case analysis included the aforementioned inputs as well as a 100 years' time horizon, societal perspective and cost-utility analysis. The ICER was calculated with the formula:

$$ICER=\;\frac{Costs_{quadrivalent}-Costs_{trivalent}}{QALYs_{quadrivalent}-QALYs_{trivalent}}$$

Although a one-year time horizon does not allow LYs or QALYs gained by alternative interventions to be calculated, first year results were reported in terms of cases avoided and costs, in order to facilitate comparisons with other assessments.

To assess the robustness of the results, two sensitivity analyses were performed: a one-way sensitivity analysis to determine which variable has individually the greatest impact on cost-effectiveness results, and a probabilistic sensitivity analysis which assessed the level of uncertainty of the variables in combination within the model. The probabilistic sensitivity analysis was performed using Monte Carlo simulations with 1,000 iterations, each selecting the input parameter values from a probability distribution. The representation of the probabilistic sensitivity analysis is presented as a cost-effectiveness acceptability curve, showing the probability of QIV being cost-effective.

## Abbreviations

ICER: Incremental cost-effectivess ratio
LY: Life year
QALY: Quality-adjusted life year
QIV: Quadrivalent influenza vaccine
NHS: National Health System
TIV: Trivalent influenza vaccine
WHO: World Health Organization
